# Role of gut microbiota‐derived metabolites in neurodegenerative disorders involving protein misfolding and C9orf72 expansion associated with dementia.

**DOI:** 10.1002/alz70859_107287

**Published:** 2025-12-26

**Authors:** Giulio Maria Pasinetti, Kenjiro Ono, Tom Lloyd, Nicole Less

**Affiliations:** ^1^ Icahn School of Medicine at Mount Sinai, New York, NY USA; ^2^ James J. Peters Veterans Affairs Medical Center, Bronx, NY USA; ^3^ Kanazawa University Graduate School of Medical Sciences, Kanazawa, Ishikawa Japan; ^4^ tlloyd4@jhmi.edu, Baltimore, MD USA; ^5^ Icahn School of Medicine at Mount Sinnai, New York, NY USA

## Abstract

**Background:**

There is growing evidence that in many neurodegenerative disorders, cell‐to‐cell transmission of a pathological, misfolded protein, such as misfolding of α‐synuclein (α‐syn) in Parkinson’s disease (PD), may be a vehicle for the spreading of pathology throughout the brain. This misfolded protein, or seed, further induces misfolding of native proteins within the cell. Pathological misfolded proteins may exist in diverse conformations with distinct cellular and biochemical properties. We investigate whether microbiota‐derived metabolites may help attenuate the misfolding of α‐syn and thereby promote resilience against PD phenotypes. We identified six biologically available gut microbiota‐derived compounds (GMP10, GMP11, GMP26, GMP28, GMP39, and GMP44) for investigation.

**Method:**

Using independent in vitro protein aggregation assays (e.g., photo‐induced cross‐linking of unmodified proteins assay, thioflavin‐T, fluorescence assay, and electron microscopy), we demonstrated that three of the compounds (GMP26, GMP44, GMP28), potently inhibit aggregations of monomeric α‐syn (or monomeric β‐amyloid peptides) into neurotoxic protein aggregates, in vitro.

**Result:**

Based on evidence linking the c9orf72 gene with expansions of GGGGCC hexanucleotide repeats and PD, as well as amyotrophic lateral sclerosis (ALS) and frontotemporal dementia (FTD), we continue to test the neuroprotective ability of our compounds in vivo using a Drosophila model with overexpression of GGGGCC hexanucleotide repeats. Overexpression of 30 GGGGCC repeats in the Drosophila eye causes age‐dependent photoreceptor neurodegeneration. We treated Drosophila by mixing individual test compounds into the food. We found all six compounds significantly suppressed eye degeneration at 10 µM, with compounds GMP26 and GMP11 almost completely suppressing the eye phenotype. The comparative efficacy of the six compounds are GMP26 = GMP11 > GMP39 > GMP10 > GMP44 > GMP28.

**Conclusion:**

Outcomes from our studies link gut microbiota with mechanisms underlying PD and suggest the feasibility of developing GMP26 as a means to simultaneously target both α‐syn misfolding and C9orf72 expansion to increase the likelihood of therapeutic efficacy in PD, ALS, FTD patients with C9orf72 expansion.

